# Prevalence and treatment patterns of erectile dysfunction and hypogonadism in men with spina bifida: a retrospective study

**DOI:** 10.3389/fruro.2025.1500839

**Published:** 2025-03-13

**Authors:** Nyemkuna Fortingo, Manpreet Sandhu, Garrick Greear, Tung-Chin Hsieh, Joshua Horns, Rupam Das, Jim Hotaling, Yahir Santiago-Lastra

**Affiliations:** ^1^ Division of Urology, Virginia Commonwealth University Health System, Richmond, VA, United States; ^2^ School of Medicine, University of California, San Diego, San Diego, CA, United States; ^3^ The Urology Center of Colorado, Denver, CO, United States; ^4^ Department of Urology, University of California, San Diego, La Jolla, CA, United States; ^5^ Division of Urology, University of Utah, Salt Lake City, UT, United States

**Keywords:** spina bifida (SB), erectile dysfunction, hypogonadism, health disparities, myelomeningocele, sexual health

## Abstract

**Objectives:**

To characterize the estimated prevalence and treatment patterns of erectile dysfunction and hypogonadism in men with spina bifida through a large claims database.

**Methods:**

This retrospective claims study used the MarketScan^®^ databases from 2008 to 2017 to compare prevalence estimates for erectile dysfunction and hypogonadism in men with spina bifida to those in men without spina bifida and to describe treatment patterns in this cohort.

**Results:**

The estimated prevalence of erectile dysfunction and hypogonadism in men with spina bifida was 7.83% and 7.71%, respectively. Men with spina bifida exhibit high rates of smoking and metabolic comorbidities but are diagnosed less frequently with erectile dysfunction when controlling for age and metabolic comorbidities than men without spina bifida. Most men with spina bifida and erectile dysfunction (66.6%) or hypogonadism (77.4%) do not receive treatment. However, a diagnosis of spina bifida did not appear to affect the likelihood of treatment for either condition on multivariate analysis.

**Conclusions:**

Men with spina bifida are known to be at high risk for erectile dysfunction but may be diagnosed or treated less frequently than age and disease-matched men without spina bifida. Hypogonadism is diagnosed more frequently in men with spina bifida, which is a new finding that warrants further investigation. Most men with SB and either ED or HG do not receive treatment. The results suggest there is potential for improving care delivery for sexual health issues in men with spina bifida.

## Introduction

Spina bifida (SB) is among the most common disabling congenital disorders in the United States, with an estimated national prevalence of 3.5 per 10,000 live births (1). Men with SB have many chronic health and occupational needs, which may result in undertreatment of sexual health issues by both patient and provider. Despite the recognized importance of sexual health, little is known about the diagnosis and management of erectile dysfunction (ED) and hypogonadism (HG) in this population.

Observational data indicates that erectile dysfunction (ED) in the SB population is highly prevalent (32 to 94%) and often responsive to phosphodiesterase-5 (PDE5) inhibitor therapy, yet sexually active men with SB rarely receive treatment (2, 3). Despite this high prevalence, treatment utilization in men with SB has yet to be quantified on a large scale. One possible reason for underdiagnosis and treatment may be due to physicians’ implicit bias about sexuality in patients with physical disability. Often there is a societal assumption that people with physical disabilities are incapable of sexual activity or lack sexual interest (4).

Similarly, the burden of hypogonadism (HG) in the SB population also remains largely unknown. Small case series have provided conflicting data on the burden of this disease in men with SB, though more robust descriptions are needed (5, 6). Furthermore, the rate of testosterone supplemental therapy (TST) in this population has never been reported, representing a critical gap in the literature.

The absence of high-volume data to guide care for these patients was noted in the most recent iteration of the SB healthcare guidelines (supported by the Spina Bifida Association) (7). These guidelines acknowledged the lack of formal recommendations on men’s health issues in SB, further emphasizing the need for research in this area. Given the paucity of registries that would enable large granular research into these sexual health topics, there remains an unmet need for robust, population-level data to better characterize these sexual health concerns.

To address this gap, our study employed a large insurance claims database to quantify the burden of ED and HG in men with SB and to evaluate the rates of treatment utilization among a more realistic and generalizable population. We hypothesized that men with SB would have higher rates of ED and HG compared to the general adult male population, and a lower rate of treatment utilization. In addition, we hypothesize a diagnosis of SB may impact treatment utilization for ED and HG in two aspects: 1) the likelihood of receiving treatment overall, and 2) the differences in treatment types among SB patients with varying disease severity.

## Materials and methods

This study was a cross-sectional observational study based on an analysis of de-identified insurance claims data from 2008 to 2017 in the MarketScan^®^ (MS) research databases, which are pooled from large employers, managed care organizations, hospitals, and public organizations. The MS databases contain medical and prescription claims for US individuals who have employer-sponsored health insurance, including Medicare supplemental coverage.

All men in Market Scan aged 18 years and over in the general population and those with a diagnosis of SB were identified for analysis. Associated hydrocephalus or tethered cord syndrome was queried for patients with SB. The earliest date (if any) of ED and HG was found using ICD-9-CM or ICD-10-CM diagnostic codes, excluding patients with congenital hormonal disorders. Each patient’s region, employment status, data type, insurance plan type, and specific metabolic comorbidities or risk factors were assessed. Therapy utilization was assessed using National Drug Codes for pharmacotherapies for ED or HG and Current Procedural Terminology (CPT) codes for penile prosthesis. **Supplementary Table 1** details specific inclusion and exclusion diagnostic and therapeutic codes.

Prevalence comparisons for ED and HG between the SB and general populations were computed. Due to high rates of overdispersion in the initial Poisson model, a negative binomial regression of the count of ED or HG patients against region, age group, SB, number of risk factors, and year was performed. The presence of HG was included as an additional covariate in the ED prevalence model. A three-way interaction between SB, age group, and the number of risk factors was tested to assess the modulating effect of SB on ED/HG prevalence within various age/risk factor strata. The total number of people in the at-risk population was included as an offset (**Supplementary Table 2**). Separate models were calculated for ED and HG.

An ANOVA was used on the ED and HG models to estimate whether the interaction of SB, age, and other relevant risk factors explained the observed variation. To more directly investigate the modulating effect of SB on age/risk factor influence of ED/HG prevalence, a series of reduced/stratified negative binomial models were calculated. The reduced model included risk factor, region, and year as cofactors (as well as HG for the ED model) while excluding age and SB. A series of models were run within each risk factor stratum where SB was re-included as a cofactor. The incidence rate ratios for ED and HG within specific metabolic risk factor and age group strata between the general population and the SB population were reported.

Variation in ED and HG treatment utilization was evaluated in two ways: 1) whether SB affected the likelihood a person would receive treatment, and 2) whether there was variation among SB patients in the form of treatment they received. For the first analysis, a Cox proportional hazards model for time from diagnosis to treatment was calculated, with age group, region, year of diagnosis, number of risk factors, and presence of SB as cofactors. Patients were censored at the time they dropped out of the MarketScan database (median follow-up time for censored ED patients = 759 days, HG patients = 493 days). For the second analysis, SB patients diagnosed with ED or HG were categorized into the form of treatment they received and Chi-square tests were applied to evaluate variation in treatment type across several clinical and demographic parameters. No adjustment was made for multiple comparisons. **Supplementary Table 1** lists the specific treatments considered in these analyses. All analyses were completed in R (version 4.0.2, R Core Team).

## Results

A total of 59,554,599 men over the age of 18 years were identified in MarketScan over the years 2008 to 2017. Of these, there were 2,476,609 (4.16%) men diagnosed with ED and 1,892,607 (3.18%) diagnosed with HG. There were 11,548 (0.019%) men with SB, of which 904 (7.83%) men had ED, 890 (7.71%) men had HG, and 262 (2.27%) had both ED and HG (**Table 1**). Men with SB showed a higher rate of metabolic comorbidities and risk factors, including smoking, obesity, hypertension, diabetes, hyperlipidemia, and vascular and ischemic heart disease, compared to the general population. This trend was also evident among men diagnosed with SB and either ED or HG as compared to men with SB alone. The proportion of men with SB who were revealed to have 3 or more metabolic risk factors was 48.2% among those diagnosed with ED and 50.2% among those diagnosed with HG (**Table 2**).

**Table 1:** Characteristics of adult men with and without SB.

Characteristic	SB No. (%)	Without SB No. (%)
Age group (years)		
18 – 34	4635 (40.5)	21,735,248 (36.5)
35 – 44	2345 (20.5)	11,276,955 (19.0)
45 – 54	2050 (17.9)	11,418,105 (19.2)
55 – 64	1616 (14.1)	10,206,269 (17.1)
65 – 74	503 (4.4)	3,007,386 (5.1)
≥75	295 (2.6)	1,899,205 (3.2)
Risk factors		
Smoking	1971 (17.2)	4,315,417 (7.3)
Obesity	1644 (14.4)	3,553,523 (6.0)
Hypertension	4810 (42.0)	14,904,537 (25.0)
Diabetes	1595 (13.9)	5,577,275 (9.4)
Hyperlipidemia	4323 (37.8)	15,857,172 (26.6)
Peripheral vascular disease	1819 (15.9)	2,435,797 (4.1)
Cerebrovascular disease	1396 (12.2)	2,254,491 (3.8)
Ischemic heart disease	1301 (11.4)	4,134,317 (6.9)
No. of risk factors		
0	3716 (32.2)	35,797,966 (60.1)
1	2758 (23.9)	9,263,662 (15.6)
2	2034 (17.6)	6,422,247 (10.8)
3+	3040 (26.3)	8,059,293 (13.5)
Erectile dysfunction	904 (7.9)	2,476,609 (4.2)
Hypogonadism	890 (7.8)	1,892,607 (3.2)
Total	11444	59,543,168

**Table 2:** Characteristics of adult males with SB, ED, and HG.

Characteristic	SB & ED No. (%)	SB & HG No. (%)	SB overall No. (%)
Age group (years)			
18 – 34	138 (15.3)	180 (20.2)	5092 (44.1)
35 – 44	211 (23.3)	237 (26.6)	2364 (20.5)
45 – 54	240 (26.6)	241 (27.1)	1993 (17.3)
55 – 64	225 (24.9)	182 (20.5)	1455 (12.6)
65 – 74	69 (7.6)	42 (4.7)	397 (3.4)
≥75	21 (2.3)	8 (0.9)	247 (2.1)
Risk factors			
Smoking	229 (25.3)	197 (22.1)	1986 (17.2)
Obesity	208 (23.0)	264 (29.7)	1665 (14.4)
Hypertension	559 (61.8)	580 (65.2)	4844 (42.0)
Diabetes	215 (23.8)	214 (24.0)	1610 (13.9)
Hyperlipidemia	577 (63.8)	588 (66.1)	4366 (37.8)
Peripheral vascular disease	212 (23.5)	165 (18.5)	1836 (15.9)
Cerebrovascular disease	167 (18.5)	165 (18.5)	1407 (12.2)
Ischemic heart disease	186 (20.6)	177 (19.9)	1320 (11.4)
No. of risk factors			
0	104 (11.5)	112 (12.6)	3716 (32.2)
1	171 (18.9)	150 (16.9)	2758 (23.9)
2	193 (21.4)	181 (20.3)	2034 (17.6)
3+	436 (48.2)	447 (50.2)	3040 (26.3)
Hydrocephalus	344 (38.1)	391 (43.9)	4671 (40.5)
Tethered cord	61 (6.8)	60 (6.7)	570 (4.9)
Bladder management			
Indwelling catheter	20 (2.2)	12 (1.4)	247 (2.1)
Intermittent catheter	120 (13.3)	89 (10.0)	1705 (14.8)
External catheter	9 (1)	8 (0.9)	95 (0.8)
None or unknown	771 (85.3)	791 (88.9)	9664 (83.7)
Total	904	890	11548

**Figure 1:**
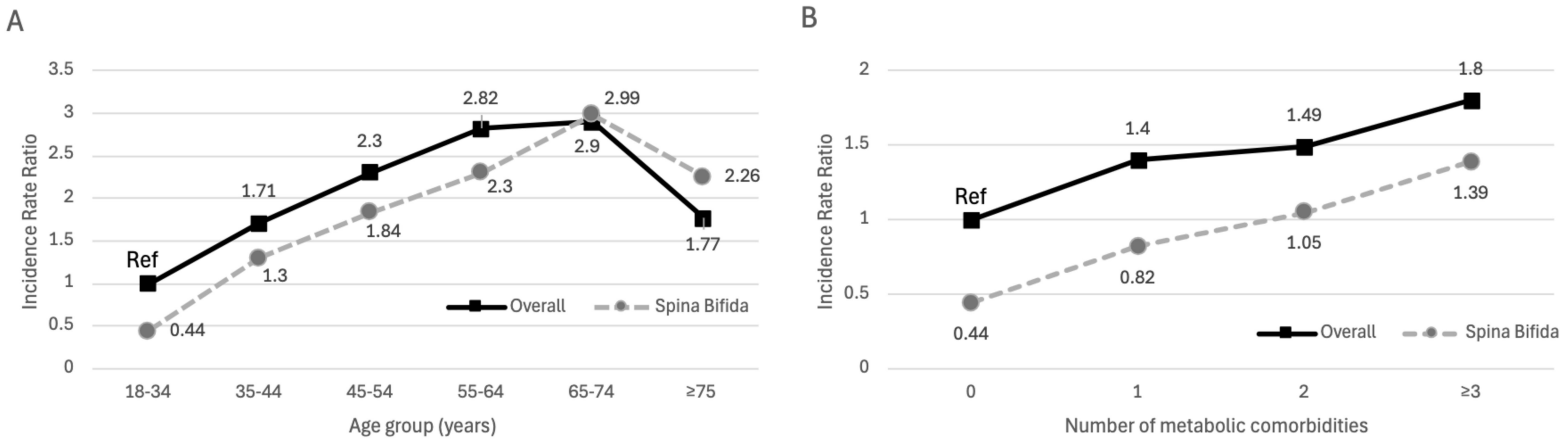
Panel **(A)** IRR for ED across age groups. Panel **(B)** IRR for ED across number of metabolic risk factors.

**Figure 2:**
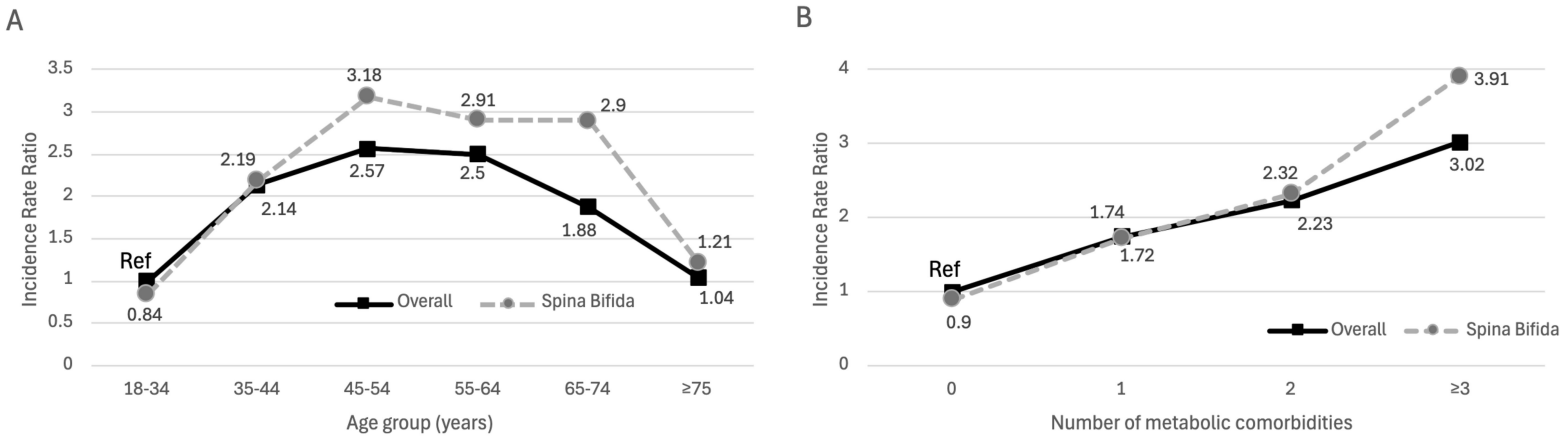
Panel **(A)** IRR for HG across age groups. Panel **(B)** IRR for HG across number of metabolic risk factors.

There was a significant three-way interaction between SB, age group, and number of risk factors for ED prevalence (p < 0.001). Compared to the adult male population without SB, when controlling for age or number of comorbid metabolic risk factors, a lower incidence rate ratio was observed for ED within the SB population across all groups except in the 65-74 years and ≥75 years age groups. In these age groups, the incidence rate ratio was similar in the 65-74 years age group and higher in men with SB in the ≥75 years age group (**Figure 1**). HG prevalence was higher in the adult male population with SB in the 45-74 years age group as compared to men without SB, and similar in both populations across risk factor number. There was no significant interaction (p = 0.198) between SB, age group, and risk factor number in HG prevalence (**Figure 2**).

Of the 904 men with SB and ED, 602 (66.6%) did not receive treatment. In a multivariate analysis controlling for region, age group, number of metabolic risk factors, year of index diagnosis, plan type, and employment status, a diagnosis of SB did not independently increase or decrease the likelihood of receiving treatment for ED relative to the general population (HR 0.992; 95% CI 0.884 – 1.113, p = 0.888) (**Supplementary Table 3**). The most common form of treatment among the SB population was a PDE5 inhibitor (n = 289, 32.0%). Only a very small proportion of men with SB received intraurethral alprostadil (n = 3, 0.33%), intracavernosal injections (n = 9, 1.0%), or penile prosthesis (n = 7, 0.77%) (**Supplementary Table 4**). There was significant variation among men with SB and ED in the form of treatment they received based on employment status (*p* < 0.001).

Of 890 men with SB and HG, 689 (77.4%) did not receive treatment with TST. In a multivariate analysis controlling for region, age group, number of metabolic risk factors, year of index diagnosis, plan type, and employment status, a diagnosis of SB did not independently increase or decrease the likelihood of receiving treatment for HG relative to the general population (HR 1.039; 95% CI 0.934 – 1.155) (**Supplementary Table 5**). The most common form of treatment was injectable testosterone (n = 186, 20.9%), followed by topical testosterone (n = 16, 1.8%). There were no records of men with SB receiving clomiphene citrate. There was significant variation among men with SB and HG in the form of treatment they received based on age group (p < 0.001), region (p = 0.04), and employment status (p < 0.001) (**Supplementary Table 6**).

## Discussion

Using a large medical claims database, this study estimated the prevalence of ED in the insured adult male SB population to be 7.83%, compared to 4.16% among adult men without SB. The estimated rate of ED in the adult male population in this study is generally consistent with prior published data validated in large claims databases: 5.6% (MarketScan, 2009 - 2014) and 6.9% (Humedica EHR, 2007 – 2014) (8). The incidence rates for ED in adult men and adult men with SB were compared. Although the unadjusted rate of ED diagnosis in men with SB was higher than in the general population, the magnitude of the difference was much less than was anticipated, especially in the context of the high rates reported in prior cohort studies of men with SB. Among prior cohort studies, the prevalence of ED ranges from 32% to 94% and correlates with penile sensation, level of dysraphism, and ambulatory status (2, 9–11). One study that objectively assessed erectile function by nocturnal penile tumescence recording displayed abnormal nocturnal erections in 13 of 15 (87%) men with SB (12). Furthermore, adjusting for age group and number of comorbidities exacerbated this discrepancy, such that ED was diagnosed less frequently in men with SB than in men without SB.

Although the SB group had a higher prevalence of metabolic risk factors, which are typically linked to higher rates of ED and HG, our findings did not align with this expected trend. The lower-than-expected prevalence of ED in men with SB in this study, compared to prior reports, may raise the possibility of underdiagnosis in this at-risk group. This gap may also be explained by limitations of the database itself, with selection bias toward patients with less severe forms of SB. Given the employer-based insurance coverage included within this dataset, the subset of men with SB captured in MarketScan is likely to represent a “best-case scenario” for resources. The use of a private insurance database, in a population that primarily utilizes public insurance, may limit the representation of spina bifida patients with less favorable outcomes or greater socioeconomic disparities. Additionally, those on private insurance often have better continence and ambulatory abilities (13). Therefore, this subset may have less neurologic impairment compared to prior historical cohort studies and possibly be at lower risk for ED.

This study underscores the impact of data source selection on prevalence research, as the choice of data sources can significantly influence our understanding of disease prevalence in at-risk populations. Relying on private insurance datasets that primarily reflect individuals with better access to resources may obscure the true prevalence of erectile dysfunction (ED) and hypogonadism in these populations, leading to an overly optimistic view of disease prevalence. To address this issue, future research could consider utilizing institutional series, which would potentially provide a more accurate representation of prevalence and health outcomes related to ED and hypogonadism in patients with spina bifida.

Still, we demonstrate that compared with the general population, men with SB in this cohort have significantly more risk factors for ED (**Table 1**). The proportion of men with SB who are diagnosed with 3 or more risk factors is nearly double the general population, and the rate of smoking is more than twice the general population. In light of the increased number of risk factors, we expected a higher burden of ED than was observed, even in the absence of any neurologic consideration. The nature of the MarketScan database does not allow us to establish the cause for this finding. Our findings suggest that further research is needed to identify whether there is an unmet need in achieving comprehensive care for adult men with SB. At a minimum, this analysis can inform counseling of men with SB with regards to smoking cessation and treatment of metabolic comorbidities and is a call to action for improved care focused on the sexual and reproductive needs of men with SB.

Among men with SB in our study who were diagnosed with ED, approximately 66% did not receive treatment, and only 2.1% received treatment other than PDE5 inhibitors (**Supplementary Table 4**). This treatment proportion is somewhat higher than estimates for the general population derived from large claims databases (74.6% “untreated”, IMS Health, 2011) (14). The low utilization of treatments such as intracavernosal injection therapy or penile prosthesis may either reflect the high efficacy of PDE5 inhibitors in this population or a lower propensity for providers to offer or seek these therapies among men with SB. Additionally, prior studies have shown that treatment for ED is often not covered in health benefits packages offered by employer-based health insurance plans, creating a financial deterrent to treatment of ED (15). The finding of significant variation in treatment modality with respect to employment status is interesting and may correlate with high-functioning status, but low overall number in each category limits broader interpretation of this analysis.

In the present study, the prevalence of HG in men with SB is estimated to be 7.71%, compared to 3.18% in the overall adult male population. This is a new, large-scale finding, as only one prior study has reported on testosterone levels in a small number of men with SB, concluding that the majority exhibited normal levels (6). More specifically, HG was diagnosed more frequently in men with SB than the general population in the 45-54, 55-64, and 65-74 years age groups. HG was also diagnosed more frequently in the SB population when 3 or more metabolic risk factors were present. Among men with SB and HG, 77.4% did not receive treatment. Despite the paucity of data on HG in men with SB, there are several small studies investigating azoospermia and fertility, suggesting a neurologically mediated deficit in spermatogenesis in men with SB (5). Yet whether HG contributes to poor semen parameters observed in men with spinal dysraphism is largely undescribed. Our finding of an increased rate of HG in men with SB has several possible explanations. Aside from the possibility of intrinsic pathology specific to SB, there is a high rate of metabolic comorbidities (i.e. those linked to metabolic syndrome) in the SB population, which dispose men toward low testosterone. These risk factors are often modifiable, which highlights an opportunity for counseling and greater coordination of care for these men. Men with SB who exhibit symptomatic hypogonadism may benefit from TST, which may also improve metabolic parameters (16–18). Additionally, although spinal insults play a larger role in the ED seen in patients with SB, it can also be of a neurovascular etiology, thus addressing and treating hypogonadism can have some small clinical benefit (19, 20).

Strengths of this study include the use of a large medical claims database, which improves the ability to capture observational data for patients with a rare condition. These databases are generated from service-level claims and prescriptions originating from a wide array of sources, including employers, health plans, and state health agencies.

This study has several limitations. There are multiple challenges related to claims-based data, accuracy of coding, and generalizability of results to men with insurance types not captured in these databases. As stated previously, there is likely a selection bias in the subset of the SB population captured in MarketScan due to the nature of the data sources. In particular, this dataset primarily includes individuals with employer-based private insurance, which may not fully represent the broader SB population, especially those with greater disease burden who rely on public insurance. Additionally, in the SB population, there was insufficient detail in diagnostic coding to evaluate the effect of the level of dysraphism and the bladder management method could not be reliably discerned via durable medical equipment codes. However, by using the presence of hydrocephalus or tethered cord as proxies for greater neurologic impairment, we did not identify a noteworthy difference in the prevalence of ED in men with SB, regardless of the presence of these conditions (**Table 2**). With respect to the analysis of hypogonadism in men with SB, several other potential risk factors (undescended testis, renal failure, steroid use, and opioid use, etc.) could not be assessed within the scope of this study but warrant further investigation. Moreover, this study relies on the accuracy of hypogonadism diagnoses as documented by clinicians rather than a standardized biochemical definition, which may introduce variability in case identification.

Despite these potential weaknesses, this study provides an important objective assessment of sexual health disorders in a vulnerable and poorly characterized cohort, highlighting key areas for further research.

## Conclusion

Prior cohort studies have reported a high prevalence of ED in men with SB. In this analysis of a large claims database, the observed prevalence of ED was lower than previously documented, which may reflect differences in study populations or potential underdiagnosis in this at-risk group. Additionally, most men with SB and ED did not receive documented treatment. The prevalence of HG also appeared higher in men with SB compared to those without SB, which may be associated with a higher burden of metabolic comorbidities and risk factors in this population. However, the nature of the dataset limits our ability to determine underlying causes for these patterns. The low rate of treatment for HG in men with SB warrants further exploration. Future research is needed to better characterize the factors contributing to these findings and to assess potential gaps in the diagnosis and management of sexual health conditions in men with SB.

## Data Availability

The raw data supporting the conclusions of this article will be made available by the authors, without undue reservation.

## References

[1] Parker SE Mai CT Canfield MA Rickard R Wang Y Meyer RE. Updated National Birth Prevalence estimates for selected birth defects in the United States, 2004-2006. Birth Defects Res A Clin Mol Teratol. (2010) 88:1008–16. doi: 10.1002/bdra.20735 20878909

[2] Shiomi T Hirayama A Fujimoto K Hirao Y. Sexuality and seeking medical help for erectile dysfunction in young adults with spina bifida. Int J Urol. (2006) 13:1323–6. doi: 10.1111/j.1442-2042.2006.01559.x 17010012

[3] Palmer JS Kaplan WE Firlit CF. Erectile dysfunction in patients with spina bifida is a treatable condition. J Urol. (2000) 164:958–61. doi: 10.1097/00005392-200009020-00009 10958716

[4] Braathen SH Carew MT Chiwaula M Rohleder P. Physical disability and sexuality, some history and some findings. In: Hunt X Braathen SH Chiwaula M Carew MT Rohleder P Swartz L, editors. Physical disability and sexuality: stories from South Africa. Palgrave Macmillan, Cham: Springer International Publishing (2021). p. 27–51. doi: 10.1007/978-3-030-55567-2_2

[5] Hultling C Levi R Amark SP Sjöblom P. Semen retrieval and analysis in men with myelomeningocele. Dev Med Child Neurol. (2000) 42:681–4. doi: 10.1017/s0012162200001250 11085296

[6] Decter RM Furness PD Nguyen TA McGowan M Laudermilch C Telenko A. Reproductive understanding, sexual functioning and testosterone levels in men with spina bifida. J Urol. (1997) 157:1466–8. doi: 10.1016/S0022-5347(01)65025-0 9120984

[7] Wiener JS Frimberger DC Wood H. Spina bifida health-care guidelines for men’s health. Urology. (2018) 116:218–26. doi: 10.1016/j.urology.2018.01.005 29545051

[8] Mulhall JP Luo X Zou KH Stecher V Galaznik A. Relationship between age and erectile dysfunction diagnosis or treatment using real-world observational data in the USA. Int J Clin Pract. (2016) 70:1012–8. doi: 10.1111/ijcp.12908 PMC5540144 28032424

[9] Gamé X Moscovici J Gamé L Sarramon JP Rischmann P Malavaud B. Evaluation of sexual function in young men with spina bifida and myelomeningocele using the International Index of Erectile Function. Urology. (2006) 67:566–70. doi: 10.1016/j.urology.2005.09.014 16504267

[10] Lee NG Andrews E Rosoklija I Logvinenko T Johnson EK Oates RD. The effect of spinal cord level on sexual function in the spina bifida population. J Pediatr Urol. (2015) 11:142. doi: 10.1016/j.jpurol.2015.02.010 25864616

[11] Roth JD Misseri R Cain MP Szymanski KM. Mobility, hydrocephalus and quality of erections in men with spina bifida. J Pediatr Urol. (2017) 13:264. doi: 10.1016/j.jpurol.2016.12.004 28111207

[12] Sandler AD Worley G Leroy EC Stanley SD Kalman S. Sexual function and erection capability among young men with spina bifida. Dev Med Child Neurol. (1996) 38:823–9. doi: 10.1111/j.1469-8749.1996.tb15117.x 8810714

[13] Schechter MS Liu T Soe M Swanson M Ward E Thibadeau J. Sociodemographic attributes and spina bifida outcomes. Pediatrics. (2015) 135:e957–964. doi: 10.1542/peds.2014-2576 PMC4536568 25780069

[14] Frederick LR Cakir OO Arora H Helfand BT McVary KT. Undertreatment of erectile dysfunction: claims analysis of 6.2 million patients. J Sex Med. (2014) 11:2546–53. doi: 10.1111/jsm.12647 25059314

[15] Burnett AL Rojanasarot S Amorosi SL. An analysis of a commercial database on the use of erectile dysfunction treatments for men with employer-sponsored health insurance. Urology. (2021) 149:140–5. doi: 10.1016/j.urology.2020.11.051 33309705

[16] Yassin A Haider A Haider KS Caliber M Doros G Saad F. Testosterone therapy in men with hypogonadism prevents progression from prediabetes to type 2 diabetes: eight-year data from a registry study. Diabetes Care. (2019) 42:1104–11. doi: 10.2337/dc18-2388 30862651

[17] Malkin CJ Jones TH Channer KS. The effect of testosterone on insulin sensitivity in men with heart failure. Eur J Heart Fail. (2007) 9:44–50. doi: 10.1016/j.ejheart.2006.04.006 16828341

[18] Jones TH Saad F. The effects of testosterone on risk factors for, and the mediators of, the atherosclerotic process. Atherosclerosis. (2009) 207:318–27. doi: 10.1016/j.atherosclerosis.2009.04.016 19464009

[19] Alwani M Yassin A Talib R Al-Qudimat A Aboumarzouk O Al-Zoubi RM. Cardiovascular disease, hypogonadism and erectile dysfunction: early detection, prevention and the positive effects of long-term testosterone treatment: prospective observational, real-life data. Vasc Health Risk Manage. (2021) 17:497–508. doi: 10.2147/VHRM.S309714 PMC8403087 34465997

[20] Shabsigh R. Testosterone therapy in erectile dysfunction and hypogonadism. J Sex Med. (2005) 2:785–92. doi: 10.1111/j.1743-6109.2005.00139.x 16422803

