# Supplementary material for: Prevalence and treatment patterns of erectile dysfunction and hypogonadism in men with spina bifida: a retrospective study
Source: Front Urol. 2025 Mar 13;5:1500839. doi: 10.3389/fruro.2025.1500839 (PMC12327303; doi:10.3389/fruro.2025.1500839)
Supplement: Supplementary file 1 [file Table1.docx]

Supplementary Table 1. List of inclusion and exclusion criteria and codes used for analysis.

| INCLUSION CRITERIA | |
| --- | --- |
| Spina bifida | ICD-9: 741.0, 741.00, 741.01, 741.02, 741.03, 741.9, 741.90, 741.91, 741.92, 741.93, 756.17  ICD-10: Q05, Q05.0, Q05.1, Q05.2, Q05.3, Q05.4, Q05.5, Q05.6, Q05.7, Q05.8, Q05.9, Q76.0 |
| Spina bifida with hydrocephalus | ICD-9: 741.0, 741.00, 741.01, 741.02, 741.03  ICD-10: Q05, Q05.0, Q05.1, Q05.2, Q05.3, Q05.4 |
| Spina bifida without hydrocephalus | ICD-9: 741.9, 741.90, 741.91, 741.92, 741.93  ICD-10: Q05.5, Q05.6, Q05.7, Q05.8, Q05.9 |
| Erectile dysfunction | ICD-9: 607.84  ICD-10: N52.xx |
| Hypogonadism | ICD-9: 257.1, 257.2, 257.8, 257.9  ICD-10: E29.1, E89.5 |
| EXCLUSION CRITERIA | |
| Gender | Female |
| Congenital genetic disorders such an androgen insensitivity syndrome, indeterminate set, chromosomal abnormalities including but not limited to non-disjunction, chimera, etc. | 259.50, 259.51, 259.52, 752.7, 788.81, 758.7, 758.81, 758.89/ E34.50, E34.51, E34.52, Q56.0, Q56.1, Q56.3, Q56.4, Q98.0, Q98.1, Q98.3-98.9, Q99.0-99.2, Q99.8, Q99.9 |
| Hypothalamic-pituitary disorders including neoplasm | D35.2, D44.3, E23.0, E23.1, E23.3, E23.6, E23.7 |
| ERECTILE DYSFUNCTION THERAPIES (National Drug Codes) | |
| PDE5 inhibitors | Sildenafil:  50090-0619-08, 54569-4570-01, 54569-4570-03, 54569-4570-08, 43353-0764-02, 43353-0764-04, 43353-0764-06, 43353-0764-12, 67544-0355-02, 67544-0355-04, 67544-0355-06, 67544-0355-12, 67544-0355-52, 67544-0355-74, 67544-0356-02, 67544-0356-04, 67544-0356-06, 67544-0356-12, 67544-0356-52, 67544-0356-56, 67544-0356-64, 67544-0356-89, 63629-6316-01, 63629-6316-96, 63629-6369-01, 63629-6369-02, 63629-6369-03, 63629-7273-01, 63629-7273-02, 55154-2736-08, 43063-0256-06, 00069-4200-30, 00069-4210-30, 00069-4210-66, 00069-4220-30, 00069-4220-66, 54868-4084-00, 54868-4084-01, 54868-4084-02, 54868-4084-03, 54868-4084-04, 54868-4084-05, 54868-4706-00, 54868-4706-01, 54868-4706-02, 54868-4706-03, 54868-4706-04, 54868-4706-05, 54868-4706-06, 54868-4706-07, 54868-4784-00, 54868-4784-01, 54868-4784-02, 21695-0157-15, 21695-0157-30, 21695-0158-30, 70518-0953-00, 63539-0421-97, 63539-0422-02, 63539-0422-03  Tadalafil:  00002-4462-30, 00002-4462-34, 00002-4463-30, 00002-4464-30, 00002-4465-34, 16590-0905-10, 21695-0028-10, 54868-4968-01, 54868-4968-02, 54868-4968-03, 54868-4968-04, 54868-4968-05, 54868-4968-06, 54868-4968-07, 54868-5956-00, 55154-1832-08, 63629-4601-01, 63629-6367-01, 63629-6769-01, 63629-7145-01, 63629-7145-02, 63629-7145-03, 63629-7145-04, 66336-0566-05, 21695-0029-10, 21695-0029-16, 21695-0029-30, 43063-0236-05, 43353-0044-04, 43353-0044-08, 43353-0044-11, 43353-0044-18, 43353-0044-47, 43353-0857-02, 43353-0857-04, 43353-0857-06, 43353-0857-12, 50436-4462-01, 50436-4462-02, 50436-4463-01, 50436-4464-01, 50436-4465-01, 54868-4665-00, 54868-4665-01, 54868-4665-02, 54868-4665-03, 54868-4665-04  Vardenafil:  43353-0323-02, 43353-0323-06, 43353-0741-02, 43353-0741-04, 43353-0741-06, 43353-0741-52, 43353-0744-02, 43353-0744-06, 43353-0748-02, 43353-0748-04. 43353-0748-06, 43353-0748-12, 43353-0748-52, 43353-0748-79, 54868-4967-00, 54868-4967-01, 54868-4967-02, 54868-4967-03, 54868-4967-04, 54868-4967-05, 54868-4967-06, 54868-4984-00, 54868-4984-01, 54868-4984-02, 54868-4984-03, 54868-4984-04, 54868-4984-05, 55289-0193-06, 63629-3372-01, 63629-3372-02, 63629-3372-03, 63629-3372-04, 67544-0507-02, 67544-0507-03, 67544-0507-04, 67544-0507-06, 67544-0507-12, 67544-0507-52, 67544-0512-02, 67544-0512-04, 67544-0512-06, 67544-0512-12, 67544-0512-52, 00173-0829-13, 00173-0830-13, 00173-0830-61, 00173-0831-13, 00173-0831-61, 00173-0822-04, 54868-6333-00  Avanafil:  76299-0320-85, 76299-0320-88, 76299-0321-85, 76299-0321-88, 76299-0321-96, 76299-0322-85, 76299-0322-88, 76299-0322-96 |
| Intraurethral suppository | Alprostadil:  00037-8110-06, 00037-8110-56, 00037-8120-06, 00037-8120-56, 00037-8130-06, 00037-8130-56, 00037-8140-06, 00037-8140-56 |
| Intracavernosal therapies | Alprostadil: 00009-3701-05, 00009-5181-01, 00009-5182-01, 00009-7686-04, 63539-0121-11, 63539-0221-21, 52244-0010-06, 52244-0010-02, 52244-0020-02, 52244-0020-06, 52244-0040-02, 52244-0040-06  Papaverine: 0517-4002-25  Phentolamine: 0143-9564-01, 0143-9564-10, 75839-285-11 |
| Penile prosthesis | CPT CODES: 54400, 54401, 54405, 54410, 54411, 54416, 54417 |
| HYPOGONADISM THERAPIES | |
| Subcutaneous hormone pellet implantation (implantation of estradiol and/or testosterone pellets beneath the skin) | 11980: this code is for all types of subcutaneous or intramuscular hormone pellet implantation; thus, it must have the testosterone pellet specific code to be specific to hypogonadism.  S0189/J3490 – testosterone pellets |
| Therapeutic, prophylactic or diagnostic injection (specify substance or drug); subcutaneous or intramuscular | 96372: this code is for all types of subcutaneous or intramuscular injections, thus, to be specific for hypogonadism, it must also include one or more NDC or J-codes for testosterone replacement medications.  J1071 – injection of testosterone cypionate, 1 mg  J3121 – injection of testosterone enanthate, 1 mg  J3145 – injection of testosterone undecanoate, 1 mg |
| Testosterone formulations | 00009-0085-10, 00009-0086-01, 00009-0086-10, 00009-0347-02, 00009-0417-01, 00009-0417-02, 00009-0520-01, 00009-0520-10, 58657-0405-01, 58657-0406-01, 62559-0150-01, 62559-0149-01, 53746-0078-01, 53746-0077-01, 00115-1408-01, 00115-1408-03, 00115-1408-08, 49884-0418-72, 45802-0366-65, 45802-0281-39, 69097-0363-44, 68180-0943-11, 45802-0754-02, 49884-0510-72, 00591-3524-30, 00254-1012-11, 00603-7831-88, 00591-3216-30, 00591-3217-30, 45802-0754-01, 00591-2114-81, 00591-2921-02, 45802-0610-01, 24979-0078-15, 00591-2363-60, 43742-1242-01, 45802-0754-03, 45802-0754-65, 68382-0362-15, 16714-0967-01, 68382-0362-14, 43742-0021-01, 45802-0116-39, 44117-0002-01, 69238-1013-02, 68180-0941-11, 00591-2924-18, 43598-0304-88, 16714-0969-02, 16714-0968-02, 45802-0754-39, 00832-1120-05, 00832-1120-35, 00832-1121-42, 68382-0362-11, 43742-0677-01, 00143-9659-01, 00143-9726-01, 50090-4446-00, 62756-0015-40, 69097-0802-32, 69097-0802-37, 52536-0625-01, 76420-0065-10, 62756-0016-40, 63187-0647-10, 00591-4128-79, 00409-6557-01, 00409-6562-01, 00409-6562-20, 00409-6562-02, 00409-6562-22, 00574-0820-01, 00574-0820-10, 71205-0289-01, 50090-4147-00, 50090-0330-00, 00574-0827-10, 52536-0625-10, 69097-0537-31, 00517-1830-01, 72833-0678-00, 64980-0467-99, 69097-0536-37, 69097-0537-37, 62756-0017-40, 00574-0827-01, 76519-1210-00, 00143-9750-01 |
| RISK FACTORS / NEUROLOGIC OR FUNCTIONAL CHARACTERISTICS | |
| Diagnosis | ICD-9/10 Codes |
| Smoking | 305.1/F17.200, V15.82/Z87.891 |
| Obesity | 278.0-278.00/E66.9x; 278.01/E66.01 |
| Hypertension | 401–405 |
| Diabetes | 250.xx/ |
| Hyperlipidemia | 272.0–272.4 |
| Peripheral vascular disease | 440–443 |
| Cerebrovascular disease | 430–438 |
| Ischemic heart disease | 410–414 |
| Tethered cord syndrome | 742.59, Q06.8 |
| Intermittent catheterization | A4351, A4352, A4353^a^ |
| Indwelling catheter | A4336, A4338, A4340, A4344, A4346^a^ |
| External catheter | A4347, A4348, A4349^a^ |
| ^a^ Healthcare Common Procedure Coding System | |
