# Supplementary material for: Prevalence and treatment patterns of erectile dysfunction and hypogonadism in men with spina bifida: a retrospective study
Source: Front Urol. 2025 Mar 13;5:1500839. doi: 10.3389/fruro.2025.1500839 (PMC12327303; doi:10.3389/fruro.2025.1500839)
Supplement: Supplementary file 2 [file Table2.docx]

Supplementary Table 2. Number of “at-risk” patients over time.

| ED | | HG | |
| --- | --- | --- | --- |
| Month | At-risk (n) | Month | At-risk (n) |
| 0 | 143794 | 0 | 324169 |
| 1 | 153341 | 1 | 148256 |
| 2 | 93202 | 2 | 85945 |
| 3 | 67208 | 3 | 65762 |
| 4 | 52586 | 4 | 58170 |
| 5 | 46459 | 5 | 54527 |
| 6 | 43627 | 6 | 50391 |
| 7 | 40623 | 7 | 50256 |
| 8 | 36747 | 8 | 45797 |
| 9 | 36766 | 9 | 46786 |
| 10 | 35579 | 10 | 44861 |
| 11 | 36675 | 11 | 48785 |
| 12 | 51600 | 12 | 42052 |
| 13 | 58819 | 13 | 29377 |
| 14 | 61910 | 14 | 30790 |
| 15 | 53638 | 15 | 27816 |
| 16 | 53524 | 16 | 27519 |
| 17 | 54046 | 17 | 28126 |
| 18 | 48139 | 18 | 25155 |
| 19 | 51669 | 19 | 27039 |
| 20 | 48027 | 20 | 25421 |
| 21 | 49783 | 21 | 26205 |
| 22 | 47953 | 22 | 25272 |
| 23 | 50453 | 23 | 26456 |
| 24 | 41363 | 24 | 22092 |
| 25 | 33605 | 25 | 15826 |
| 26 | 35537 | 26 | 17309 |
| 27 | 31722 | 27 | 15685 |
| 28 | 31264 | 28 | 15206 |
| 29 | 30448 | 29 | 15559 |
| 30 | 29261 | 30 | 14919 |
| 31 | 30306 | 31 | 15324 |
| 32 | 28874 | 32 | 14876 |
| 33 | 30224 | 33 | 15278 |
| 34 | 29075 | 34 | 14765 |
| 35 | 30281 | 35 | 15596 |
| 36 | 25626 | 36 | 13048 |
| 37 | 21355 | 37 | 9619 |
| 38 | 23055 | 38 | 10723 |
| 39 | 20387 | 39 | 9771 |
| 40 | 20565 | 40 | 9599 |
| 41 | 21315 | 41 | 10189 |
| 42 | 18769 | 42 | 9115 |
| 43 | 20331 | 43 | 9883 |
| 44 | 19265 | 44 | 9303 |
| 45 | 20396 | 45 | 10013 |
| 46 | 19521 | 46 | 9545 |
| 47 | 20783 | 47 | 10258 |
| 48 | 17529 | 48 | 8285 |
| 49 | 14682 | 49 | 6509 |
| 50 | 15980 | 50 | 7221 |
| 51 | 14016 | 51 | 6561 |
| 52 | 13917 | 52 | 6493 |
| 53 | 13800 | 53 | 6573 |
| 54 | 13290 | 54 | 6323 |
| 55 | 13501 | 55 | 6577 |
| 56 | 13058 | 56 | 6300 |
| 57 | 14108 | 57 | 6592 |
| 58 | 12936 | 58 | 6201 |
| 59 | 13819 | 59 | 6637 |
| 60 | 10764 | 60 | 5070 |
| 61 | 8063 | 61 | 3747 |
| 62 | 8763 | 62 | 4320 |
| 63 | 7618 | 63 | 3749 |
| 64 | 7487 | 64 | 3733 |
| 65 | 8001 | 65 | 4035 |
| 66 | 6956 | 66 | 3447 |
| 67 | 7721 | 67 | 3940 |
| 68 | 7544 | 68 | 3626 |
| 69 | 7770 | 69 | 3716 |
| 70 | 7606 | 70 | 3506 |
| 71 | 8112 | 71 | 3679 |
| 72 | 6751 | 72 | 2885 |
| 73 | 6321 | 73 | 2460 |
| 74 | 6829 | 74 | 2507 |
| 75 | 6214 | 75 | 2289 |
| 76 | 6176 | 76 | 2419 |
| 77 | 6248 | 77 | 2297 |
| 78 | 6004 | 78 | 2231 |
| 79 | 6558 | 79 | 2285 |
| 80 | 6081 | 80 | 2084 |
| 81 | 6455 | 81 | 2302 |
| 82 | 6261 | 82 | 2208 |
| 83 | 6757 | 83 | 2396 |
| 84 | 4643 | 84 | 1743 |
| 85 | 2651 | 85 | 1010 |
| 86 | 2770 | 86 | 1113 |
| 87 | 2443 | 87 | 1000 |
| 88 | 2263 | 88 | 982 |
| 89 | 2147 | 89 | 1017 |
| 90 | 1873 | 90 | 885 |
| 91 | 1996 | 91 | 961 |
| 92 | 1919 | 92 | 867 |
| 93 | 2017 | 93 | 942 |
| 94 | 1921 | 94 | 831 |
| 95 | 2010 | 95 | 867 |
| 96 | 1657 | 96 | 716 |
| 97 | 1492 | 97 | 621 |
| 98 | 1614 | 98 | 625 |
| 99 | 1629 | 99 | 623 |
| 100 | 1580 | 100 | 567 |
| 101 | 1495 | 101 | 684 |
| 102 | 1398 | 102 | 588 |
| 103 | 1445 | 103 | 574 |
| 104 | 1419 | 104 | 580 |
| 105 | 1516 | 105 | 602 |
| 106 | 1366 | 106 | 520 |
| 107 | 1449 | 107 | 609 |
| 108 | 1235 | 108 | 474 |
| 109 | 1007 | 109 | 351 |
| 110 | 1101 | 110 | 364 |
| 111 | 1135 | 111 | 349 |
| 112 | 1261 | 112 | 378 |
| 113 | 1322 | 113 | 391 |
| 114 | 1177 | 114 | 353 |
| 115 | 1231 | 115 | 324 |
| 116 | 1101 | 116 | 354 |
| 117 | 1210 | 117 | 379 |
| 118 | 1129 | 118 | 376 |
| 119 | 1094 | 119 | 448 |
| 120 | 842 | 120 | 226 |
| 121 | 496 |  |  |
| 122 | 519 |  |  |
| 123 | 232 |  |  |
| 124 | 28 |  |  |
| 125 | 2 |  |  |
