# Supplementary material for: Prevalence and treatment patterns of erectile dysfunction and hypogonadism in men with spina bifida: a retrospective study
Source: Front Urol. 2025 Mar 13;5:1500839. doi: 10.3389/fruro.2025.1500839 (PMC12327303; doi:10.3389/fruro.2025.1500839)
Supplement: Supplementary file 3 [file Table3.docx]

Supplementary Table 3. Multivariate analysis of factors associated with receiving treatment for ED.

| Characteristic | Hazard ratio [95% CI] | *p*-value |
| --- | --- | --- |
| Spina bifida |  |  |
| No | Reference |  |
| Yes | 0.992 [0.884-1.113] | 0.888 |
| Region |  |  |
| Northeast | Reference |  |
| Midwest | 0.786 [0.78-0.792] | < 0.001 |
| South | 0.707 [0.702-0.712] | < 0.001 |
| West | 0.715 [0.709-0.72] | < 0.001 |
| Other | 0.64 [0.627-0.654] | < 0.001 |
| Age group (years) |  |  |
| 18-34 | Reference |  |
| 35-44 | 1.431 [1.412-1.451] | < 0.001 |
| 45-54 | 1.568 [1.548-1.588] | < 0.001 |
| 55-64 | 1.433 [1.415-1.452] | < 0.001 |
| 65-74 | 0.981 [0.965-0.997] | 0.022 |
| 75+ | 0.68 [0.666-0.695] | < 0.001 |
| No. of metabolic risk factors |  |  |
| 0 | Reference |  |
| 1 | 1.032 [1.023-1.041] | < 0.001 |
| 2 | 1.047 [1.038-1.056] | < 0.001 |
| 3+ | 0.999 [0.991-1.007] | 0.79 |
| Diagnosis Year |  |  |
| 2008 | Reference |  |
| 2009 | 0.909 [0.899-0.918] | < 0.001 |
| 2010 | 0.863 [0.854-0.872] | < 0.001 |
| 2011 | 0.891 [0.881-0.9] | < 0.001 |
| 2012 | 0.807 [0.798-0.815] | < 0.001 |
| 2013 | 0.79 [0.781-0.798] | < 0.001 |
| 2014 | 0.793 [0.784-0.801] | < 0.001 |
| 2015 | 0.85 [0.84-0.86] | < 0.001 |
| 2016 | 0.741 [0.732-0.751] | < 0.001 |
| 2017 | 0.596 [0.588-0.605] | < 0.001 |
| Plan Type |  |  |
| Comprehensive | Reference |  |
| EPO | 1.459 [1.423-1.496] | < 0.001 |
| HMO | 1.508 [1.486-1.531] | < 0.001 |
| POS | 1.521 [1.497-1.546] | < 0.001 |
| PPO | 1.582 [1.562-1.604] | < 0.001 |
| POS with cap. | 1.537 [1.481-1.594] | < 0.001 |
| CDHP | 1.16 [1.139-1.18] | < 0.001 |
| HDHP | 1.169 [1.146-1.193] | < 0.001 |
| Missing | 1.732 [1.703-1.762] | < 0.001 |
| Employment Status |  |  |
| Active full-time | Reference |  |
| Active part-time | 1.072 [1.043-1.102] | < 0.001 |
| Early retiree | 0.914 [0.904-0.924] | < 0.001 |
| Medicare-eligible retiree | 1.119 [1.103-1.135] | < 0.001 |
| Retiree (unknown) | 0.94 [0.924-0.958] | < 0.001 |
| COBRA | 0.953 [0.918-0.989] | 0.011 |
| Long-term disability | 1.102 [1.046-1.16] | < 0.001 |
| Surviving spouse/depen. | 1.286 [1.21-1.366] | < 0.001 |
| Unknown | 0.379 [0.376-0.381] | < 0.001 |
