# Supplementary material for: Prevalence and treatment patterns of erectile dysfunction and hypogonadism in men with spina bifida: a retrospective study
Source: Front Urol. 2025 Mar 13;5:1500839. doi: 10.3389/fruro.2025.1500839 (PMC12327303; doi:10.3389/fruro.2025.1500839)
Supplement: Supplementary file 4 [file Table4.docx]

Supplementary Table 4. Treatments for erectile dysfunction in men with spina bifida.

| Characteristic | PDE5 inhibitor | Intraure- thral alprost-adil | Intracav-ernosal injection | Penile prosthesis | None | Total |
| --- | --- | --- | --- | --- | --- | --- |
| **Spina bifida overall, No. (%)** | 289 (32) | 3 (0.33) | 9 (1) | 7 (0.77) | 602 (66.59) | 904 |
| **Hydrocephalus, No. (%)** | **p = 0.14^a^** | | | | | |
| Present | 95 (27.6) | 2 (0.58) | 2 (0.6) | 3 (0.87) | 244 (70.93) | 344 |
| Not present | 195 (34.6) | 1 (0.18) | 7 (1.2) | 4 (0.71) | 361 (64.01) | 564 |
| **Tethered cord, No. (%)** | **p = 0.82^a^** | | | | | |
| Present | 22 (36.1) | 0 (0) | 1 (1.6) | 0 (0) | 38 (62.3) | 61 |
| Not present | 267 (31.7) | 3 (0.36) | 8 (1) | 7 (0.83) | 564 (66.9) | 843 |
| **Age group, No. (%)** | **p = 0.54^a^** | | | | | |
| 18-34 | 40 (29) | 0 (0) | 0 (0) | 0 (0) | 98 (71.01) | 138 |
| 35-44 | 72 (34.1) | 1 (0.47) | 1 (0.5) | 1 (0.47) | 138 (65.4) | 211 |
| 45-54 | 79 (32.9) | 1 (0.42) | 5 (2.1) | 5 (2.08) | 152 (63.33) | 240 |
| 55-64 | 75 (33.3) | 1 (0.44) | 2 (0.9) | 0 (0) | 148 (65.78) | 225 |
| 65-74 | 18 (26.1) | 0 (0) | 1 (1.5) | 1 (1.45) | 50 (72.46) | 69 |
| 75+ | 5 (23.8) | 0 (0) | 0 (0) | 0 (0) | 16 (76.19) | 21 |
| **Bladder management, No. (%)** | **p = 0.05^a^** | | | | | |
| Indwelling catheter | 6 (30) | 0 (0) | 0 (0) | 0 (0) | 14 (70) | 20 |
| Intermittent catheter | 25 (20.8) | 0 (0) | 4 (3.33) | 2 (1.67) | 89 (74.2) | 120 |
| External catheter | 2 (22.2) | 0 (0) | 0 (0) | 0 (0) | 7 (77.8) | 9 |
| None/unknown | 261 (33.9) | 3 (0.39) | 5 (0.65) | 5 (0.65) | 503 (65.2) | 771 |
| **Region, No. (%)** | **p = 0.65^a^** | | | | | |
| Northeast | 47 (30.9) | 1 (0.66) | 2 (1.3) | 1 (0.66) | 102 (67.11) | 152 |
| Midwest | 86 (39.6) | 1 (0.46) | 2 (0.9) | 3 (1.38) | 129 (59.45) | 217 |
| South | 112 (30.4) | 1 (0.27) | 3 (0.8) | 2 (0.54) | 251 (68.21) | 368 |
| West | 39 (28.1) | 0 (0) | 2 (1.4) | 1 (0.72) | 97 (69.78) | 139 |
| Other | 5 (17.9) | 0 (0) | 0 (0) | 0 (0) | 23 (82.14) | 28 |
| **Population Density, No. (%)** | **p = 0.53^a^** | | | | | |
| Urban | 243 (32.8) | 3 (0.4) | 6 (0.8) | 6 (0.81) | 489 (65.9) | 742 |
| Rural | 40 (30.1) | 0 (0) | 3 (2.3) | 1 (0.75) | 90 (67.67) | 133 |
| Unknown | 6 (20.7) | 0 (0) | 0 (0) | 0 (0) | 23 (79.31) | 29 |
| **Employment Status, No. (%)** | **p < 0.001^a^** | | | | | |
| Active Full-time | 124 (41.5) | 1 (0.33) | 4 (1.3) | 4 (1.34) | 168 (56.19) | 299 |
| Active Part-time | 0 (0) | 0 (0) | 0 (0) | 0 (0) | 5 (100) | 5 |
| Early Retiree | 18 (66.7) | 0 (0) | 0 (0) | 0 (0) | 9 (33.33) | 27 |
| Medicare-eligible retiree | 13 (39.4) | 0 (0) | 2 (6.1) | 0 (0) | 19 (57.58) | 33 |
| Retiree (unknown) | 8 (44.4) | 0 (0) | 0 (0) | 0 (0) | 10 (55.56) | 18 |
| COBRA | 2 (50) | 0 (0) | 0 (0) | 0 (0) | 2 (50) | 4 |
| Long-term disability | 3 (42.9) | 0 (0) | 1 (14.3) | 0 (0) | 4 (57.14) | 7 |
| Surviving spouse/dependent | 0 (0) | 0 (0) | 0 (0) | 0 (0) | 1 (100) | 1 |
| Other/unknown | 121 (23.7) | 2 (0.39) | 2 (0.4) | 3 (0.59) | 384 (75.29) | 510 |
| **Data Type, No. (%)** | **p = 0.54^a^** | | | | | |
| Fee for service | 230 (31.5) | 3 (0.41) | 7 (1) | 6 (0.82) | 489 (66.99) | 730 |
| Encounter | 35 (43.8) | 0 (0) | 1 (1.3) | 0 (0) | 44 (55) | 80 |
| Medicare | 23 (28.1) | 0 (0) | 1 (1.2) | 1 (1.22) | 58 (70.73) | 82 |
| Medicare encounter | 1 (8.3) | 0 (0) | 0 (0) | 0 (0) | 11 (91.67) | 12 |
| **Plan Type, No. (%)** | **p = 0.61^a^** | | | | | |
| Comprehensive | 14 (36.8) | 0 (0) | 1 (2.6) | 0 (0) | 24 (63.16) | 38 |
| EPO/PPO | 184 (31.2) | 3 (0.51) | 7 (1.2) | 4 (0.68) | 397 (67.29) | 590 |
| HMO | 36 (40) | 0 (0) | 1 (1.1) | 0 (0) | 53 (58.89) | 90 |
| POS w/wo capitation | 23 (37.7) | 0 (0) | 0 (0) | 0 (0) | 38 (62.3) | 61 |
| CDHP/HDHP | 15 (23.1) | 0 (0) | 0 (0) | 2 (3.08) | 48 (73.85) | 65 |
| Other/unknown | 17 (28.3) | 0 (0) | 0 (0) | 1 (1.67) | 42 (70) | 60 |
| ^a^ Reported p-values reflect the result of Chi-square test for significant variation | | | | | | |
