# Supplementary material for: Prevalence and treatment patterns of erectile dysfunction and hypogonadism in men with spina bifida: a retrospective study
Source: Front Urol. 2025 Mar 13;5:1500839. doi: 10.3389/fruro.2025.1500839 (PMC12327303; doi:10.3389/fruro.2025.1500839)
Supplement: Supplementary file 5 [file Table5.docx]

Supplementary Table 5. Multivariate analysis of factors associated with receiving treatment for HG.

| Characteristic | Hazard ratio [95% CI] | *p*-value |
| --- | --- | --- |
| Spina bifida |  |  |
| No | Reference |  |
| Yes | 1.039 [0.934-1.155] | 0.482 |
| Region |  |  |
| Northeast | Reference |  |
| Midwest | 1.546 [1.531-1.562] | < 0.001 |
| South | 1.614 [1.6-1.628] | < 0.001 |
| West | 1.447 [1.432-1.461] | < 0.001 |
| Other | 1.426 [1.398-1.455] | < 0.001 |
| Age group (years) |  |  |
| 18-34 | Reference |  |
| 35-44 | 1.396 [1.381-1.411] | < 0.001 |
| 45-54 | 1.371 [1.357-1.385] | < 0.001 |
| 55-64 | 1.207 [1.195-1.22] | < 0.001 |
| 65-74 | 1.077 [1.061-1.093] | < 0.001 |
| 75+ | 0.903 [0.885-0.922] | < 0.001 |
| No. of metabolic risk factors |  |  |
| 0 | Reference |  |
| 1 | 1.048 [1.039-1.057] | < 0.001 |
| 2 | 1.104 [1.094-1.113] | < 0.001 |
| 3+ | 1.132 [1.123-1.141] | < 0.001 |
| Diagnosis Year |  |  |
| 2008 | Reference |  |
| 2009 | 1.148 [1.134-1.162] | < 0.001 |
| 2010 | 1.276 [1.261-1.29] | < 0.001 |
| 2011 | 1.409 [1.394-1.424] | < 0.001 |
| 2012 | 1.458 [1.443-1.473] | < 0.001 |
| 2013 | 1.268 [1.255-1.282] | < 0.001 |
| 2014 | 1.092 [1.079-1.104] | < 0.001 |
| 2015 | 0.797 [0.786-0.808] | < 0.001 |
| 2016 | 0.793 [0.782-0.805] | < 0.001 |
| 2017 | 0.923 [0.909-0.937] | < 0.001 |
| Plan Type |  |  |
| Comprehensive | Reference |  |
| EPO | 0.974 [0.95-0.998] | 0.034 |
| HMO | 1.203 [1.184-1.223] | < 0.001 |
| POS | 1.171 [1.151-1.19] | < 0.001 |
| PPO | 1.123 [1.107-1.139] | < 0.001 |
| POS with cap. | 1.162 [1.123-1.202] | < 0.001 |
| CDHP | 0.984 [0.967-1.002] | 0.087 |
| HDHP | 0.992 [0.972-1.011] | 0.394 |
| Missing | 1.039 [1.02-1.059] | < 0.001 |
| Employment Status |  |  |
| Active full-time | Reference |  |
| Active part-time | 0.957 [0.929-0.986] | 0.004 |
| Early retiree | 0.985 [0.973-0.998] | 0.021 |
| Medicare-eligible retiree | 1.191 [1.172-1.211] | < 0.001 |
| Retiree (unknown) | 1.031 [1.009-1.053] | 0.005 |
| COBRA | 1.081 [1.041-1.123] | < 0.001 |
| Long-term disability | 1.205 [1.148-1.266] | < 0.001 |
| Surviving spouse/depen. | 1.107 [1.017-1.204] | 0.018 |
| Unknown | 0.726 [0.722-0.73] | < 0.001 |
