# Supplementary material for: Prevalence and treatment patterns of erectile dysfunction and hypogonadism in men with spina bifida: a retrospective study
Source: Front Urol. 2025 Mar 13;5:1500839. doi: 10.3389/fruro.2025.1500839 (PMC12327303; doi:10.3389/fruro.2025.1500839)
Supplement: Supplementary file 6 [file Table6.docx]

Supplementary Table 6. Treatments for hypogonadism in men with spina bifida.

| Characteristic | Intramuscular/Sub-cutaneous | Topical | None | Total |
| --- | --- | --- | --- | --- |
| **Spina bifida overall, No. (%)** | 186 (20.9) | 16 (1.8) | 689 (77.4) | 890 |
| **Hydrocephalus, No. (%)** | **p = 0.47^a^** | | | |
| Present | 78 (20) | 5 (1.3) | 309 (79) | 391 |
| Not present | 108 (21.5) | 11 (2.2) | 383 (76.3) | 502 |
| **Tethered cord, No. (%)** | **p = 0.55^a^** | | | |
| Present | 13 (21.7) | 0 (0) | 47 (78.3) | 60 |
| Not present | 173 (20.8) | 16 (1.9) | 642 (77.4) | 830 |
| **Age group, No. (%)** | **p < 0.001^a^** | | | |
| 18-34 | 23 (12.8) | 0 (0) | 157 (87.2) | 180 |
| 35-44 | 74 (31.2) | 5 (2.1) | 159 (67.1) | 237 |
| 45-54 | 50 (20.8) | 9 (3.7) | 182 (75.5) | 241 |
| 55-64 | 31 (17) | 2 (1.1) | 149 (81.9) | 182 |
| 65-74 | 6 (14.3) | 0 (0) | 36 (85.7) | 42 |
| 75+ | 2 (25) | 0 (0) | 6 (75) | 8 |
| **Bladder management, No. (%)** | **p = 0.99^a^** | | | |
| Indwelling catheter | 2 (16.7) | 0 (0) | 10 (83.3) | 12 |
| Intermittent catheter | 19 (21.4) | 2 (2.3) | 68 (76.4) | 89 |
| External catheter | 2 (25) | 0 (0) | 6 (75) | 8 |
| None/unknown | 165 (20.9) | 14 (1.8) | 613 (77.5) | 791 |
| **Region, No. (%)** | **p = 0.041^a^** | | | |
| Northeast | 20 (15.6) | 2 (1.6) | 106 (82.8) | 128 |
| Midwest | 25 (18.9) | 1 (0.8) | 107 (81.1) | 132 |
| South | 115 (25.3) | 8 (1.8) | 332 (73) | 455 |
| West | 22 (15.8) | 5 (3.6) | 112 (80.6) | 139 |
| Other | 4 (11.1) | 0 (0) | 32 (88.9) | 36 |
| **Population Density, No. (%)** | **p = 0.93^a^** | | | |
| Urban | 152 (21.1) | 14 (1.9) | 554 (76.9) | 720 |
| Rural | 28 (22.4) | 2 (1.6) | 96 (76.8) | 125 |
| Unknown | 6 (13.3) | 0 (0) | 39 (86.7) | 45 |
| **Employment Status, No. (%)** | **p < 0.001^a^** | | | |
| Active Full-time | 117 (28.8) | 13 (3.2) | 277 (68.2) | 406 |
| Active Part-time | 0 (0) | 0 (0) | 10 (100) | 10 |
| Early Retiree | 4 (13.8) | 1 (3.5) | 24 (82.8) | 29 |
| Medicare-eligible retiree | 5 (23.8) | 0 (0) | 16 (76.2) | 21 |
| Retiree (unknown) | 3 (27.3) | 0 (0) | 8 (72.7) | 11 |
| COBRA | 0 (0) | 0 (0) | 4 (100) | 4 |
| Long-term disability | 2 (66.7) | 0 (0) | 1 (33.3) | 3 |
| Surviving spouse/dependent | 0 (0) | 0 (0) | 0 (0) | 0 |
| Other/unknown | 55 (13.6) | 2 (0.5) | 349 (86) | 406 |
| **Data Type, No. (%)** | **p = 0.60^a^** | | | |
| Fee for service | 160 (20.9) | 13 (1.7) | 591 (77.4) | 764 |
| Encounter | 17 (23) | 3 (4.1) | 55 (74.3) | 74 |
| Medicare | 9 (18.8) | 0 (0) | 39 (81.3) | 48 |
| Medicare encounter | 0 (0) | 0 (0) | 4 (100) | 4 |
| **Plan Type, No. (%)** | **p = 0.84^a^** | | | |
| Comprehensive | 3 (18.8) | 0 (0) | 13 (81.3) | 16 |
| EPO/PPO | 123 (21.1) | 10 (1.7) | 449 (77.2) | 582 |
| HMO | 17 (23.6) | 3 (4.2) | 53 (73.6) | 72 |
| POS w/wo capitation | 14 (18.4) | 2 (2.6) | 60 (79) | 76 |
| CDHP/HDHP | 20 (22.5) | 1 (1.1) | 68 (76.4) | 89 |
| Other/unknown | 9 (16.4) | 0 (0) | 46 (83.6) | 55 |
| ^a^ Reported p-values reflect the result of Chi-square test for significant variation | | | | |
